# Characterising an Alternative Murine Model of Diabetic Cardiomyopathy

**DOI:** 10.3389/fphys.2019.01395

**Published:** 2019-11-14

**Authors:** Mitchel Tate, Darnel Prakoso, Andrew M. Willis, Cheng Peng, Minh Deo, Cheng Xue Qin, Jesse L. Walsh, David M. Nash, Charles D. Cohen, Alex K. Rofe, Arpeeta Sharma, Helen Kiriazis, Daniel G. Donner, Judy B. De Haan, Anna M. D. Watson, Miles J. De Blasio, Rebecca H. Ritchie

**Affiliations:** ^1^Heart Failure Pharmacology, Baker Heart and Diabetes Institute, Melbourne, VIC, Australia; ^2^Department of Diabetes, Central Clinical School, Monash University, Melbourne, VIC, Australia; ^3^School of Biosciences, The University of Melbourne, Melbourne, VIC, Australia; ^4^Oxidative Stress Laboratory, Baker Heart and Diabetes Institute, Melbourne, VIC, Australia; ^5^Preclinical Cardiology, Microsurgery and Imaging Platform, Baker Heart and Diabetes Institute, Melbourne, VIC, Australia; ^6^Department of Pharmacology and Therapeutics, The University of Melbourne, Melbourne, VIC, Australia

**Keywords:** diabetes, type 2 diabetes, diabetic cardiomyopathy, cardiac, experimental model

## Abstract

The increasing burden of heart failure globally can be partly attributed to the increased prevalence of diabetes, and the subsequent development of a distinct form of heart failure known as diabetic cardiomyopathy. Despite this, effective treatment options have remained elusive, due partly to the lack of an experimental model that adequately mimics human disease. In the current study, we combined three consecutive daily injections of low-dose streptozotocin with high-fat diet, in order to recapitulate the long-term complications of diabetes, with a specific focus on the diabetic heart. At 26 weeks of diabetes, several metabolic changes were observed including elevated blood glucose, glycated haemoglobin, plasma insulin and plasma C-peptide. Further analysis of organs commonly affected by diabetes revealed diabetic nephropathy, underlined by renal functional and structural abnormalities, as well as progressive liver damage. In addition, this protocol led to robust left ventricular diastolic dysfunction at 26 weeks with preserved systolic function, a key characteristic of patients with type 2 diabetes-induced cardiomyopathy. These observations corresponded with cardiac structural changes, namely an increase in myocardial fibrosis, as well as activation of several cardiac signalling pathways previously implicated in disease progression. It is hoped that development of an appropriate model will help to understand some the pathophysiological mechanisms underlying the accelerated progression of diabetic complications, leading ultimately to more efficacious treatment options.

## Introduction

The prevalence of diabetes is increasing at an alarming rate; recent estimations predict that 642 million adults will be affected by diabetes by 2040 (Ogurtsova et al., 2017). Importantly, diabetes patients have an increased risk of developing associated complications including nephropathy, neuropathy, retinopathy and cardiovascular disease (Adler et al., 1997; Retnakaran et al., 2006; Semeraro et al., 2015). Although diabetes confers up to a fivefold increased risk of developing heart failure, this cannot be fully attributed to the presence of hypertension and coronary heart disease (Kannel et al., 1974; Marwick et al., 2018). In fact, diabetes patients can develop a distinct form of heart failure, termed diabetic cardiomyopathy, that is characterised by an initial diastolic dysfunction in the absence of systolic dysfunction, often referred to as heart failure with preserved ejection fraction (HFpEF) (Lourenço et al., 2018; Seferović et al., 2018).

Despite being the subject of intense research, the pathophysiological mechanisms underlying the progressive degenerative changes in the heart that have limited capacity for repair and remain incompletely understood (Tate et al., 2017b). As a result, no therapeutic option to specifically treat diabetic cardiomyopathy currently exists (Marwick et al., 2018). The diabetic milieu elicits changes in several cell types in the heart, including cardiomyocytes, cardiac fibroblasts, inflammatory cells and endothelial cells. These changes promote detrimental cardiac remodelling including cardiac hypertrophy, cardiomyocyte apoptosis and myocardial fibrosis (Bugyei-Twum et al., 2016; Russo and Frangogiannis, 2016; Tate et al., 2017b; Zhou et al., 2018).

Diabetes can be split into two major subtypes; type 1 diabetes (T1DM), where an autoimmune response raised against the pancreatic β-cells impairs insulin production, and type 2 diabetes (T2DM) which is characterised by insulin resistance and often succeeded by β-cell dysfunction (Riehle and Abel, 2016). Although T1DM and T2DM are distinct at a systemic level, they share a number of similarities in terms of impact on the myocardium, sharing common structural and functional features of clinical diabetic cardiomyopathy (Bugger and Abel, 2008). In fact, several small and large experimental models of T1DM and T2DM have been utilised to study these effects.

Mice are the most widely utilised animal in experimental studies due to their short breeding cycles, a genetic similarity to humans and the availability of genetically-modified mice (Mouse Genome Sequencing Consortium et al., 2002; Riehle and Bauersachs, 2018). Several protocols have been developed to induce diabetes by taking advantage of genetics, supplemented diets, as well as chemically-induced models. The pancreatic β-cell toxin, streptozotocin (STZ), is commonly used to induce β-cell necrosis and an insulin production deficiency (Tate et al., 2016, 2017a; Prakoso et al., 2017). Although this model does not mimic the more clinically prevalent T2DM, the STZ model avoids confounding factors including obesity and impaired leptin signalling which need to be taken into account in common genetic models of T2DM, including spontaneously diabetic *db/db* and *ob/ob* mice. Recently, there has been an emergence of T2DM models incorporating low-dose STZ alongside dietary intervention, as high-fat diet alone is not enough to induce diabetes (Barrière et al., 2018; Wanrooy et al., 2018). Notably, the use of low-dose STZ and high fat diet in a rat model replicates late-stage clinical T2DM where β-cell loss is apparent (Butler et al., 2003). Therefore, this study sought to characterise the cardiac structural and functional changes in a T2DM mouse model incorporating low-dose STZ superimposed on a high-fat diet.

## Materials and Methods

### Animals

All activities involving the use of animals for research were approved by the Alfred Medical Research Education Precinct Animal Ethics Committee and were conducted according to guidelines of the National Health and Medical Research Council of Australia for animal experimentation. FVB/N mice were sourced from the Alfred Medical Research and Education Precinct Animal Services. Mice had free access to food and water and were housed at 22 ± 1°C on a 12 h light/dark cycle.

### Experimental Design

For all experiments, we have included flow charts for the reporting of animal use and analysis in preclinical studies (Supplementary Figure 1). The main aim of this study was to investigate cardiac structure and function in an experimental model of T2DM-induced cardiomyopathy. Accordingly, our primary endpoint was impact of diabetes on E/A ratio and e′/a′ ratio, markers of left ventricular (LV) diastolic function. Male 6-week-old FVB/N mice received three consecutive daily i.p. injections of STZ (55 mg/kg body weight, in 0.1 mol/l citric acid vehicle, pH 4.5; Sigma) combined with 18 weeks of high-fat diet (42% energy intake from lipids, SF04-001, Specialty Feeds) to induce T2DM. Non-diabetic mice were randomly allocated to citric acid vehicle combined with normal chow-diet. Diabetes was confirmed by measuring blood glucose every 2 weeks via the saphenous vein using a glucometer (Accu-Chek, Roche). One week prior to tissue collection, whole body composition analysis was performed using an Echo-MRI^TM^ 4-in-1 700 Analyser. Mice were placed individually into metabolic cages for 24 h in the final week of the study. Urine and plasma samples were collected for subsequent analysis. Glycated haemoglobin (HbA1c) was measured at study end using the Cobas b 101 POC system (Roche). At study end, animals received a dose of ketamine/xylazine (85/8.5 mg/kg i.p.) prior to exsanguination and rapid excision of the heart. The remainder of the LV was collected for processing or snap-frozen in liquid nitrogen and stored at −80°C for biochemical analysis.

### Intraperitoneal Glucose Tolerance Test

Intraperitoneal glucose tolerance tests were conducted 1 week prior to endpoint. Prior to IPGTT, mice were fasted for 5 h and had their baseline blood glucose level recorded. At time 0, a 25% glucose solution (4 μl/g, Baxter, Viaflex^®^) was injected via a single i.p bolus, after which blood glucose measurements were obtained via tail vein bleeds at 15, 30, 45, 60, 90 and 120 min. Area-under-the-curve (AUC) was calculated to determine the rate of glucose clearance.

### Plasma Analysis

Circulating plasma insulin (cat no. 80-INSMSU-E01, ALPCO), C-peptide (cat. no. 80-CPTMS-E01, ALPCO) and albumin (cat no. ab207620, Abcam) concentrations at endpoint were measured by mouse-specific ELISA kit, as per manufacturer’s instructions. Plasma levels of alanine aminotransferase (ALT, cat no. 20764957, Roche) and aspartate aminotransferase (AST, cat no. 20764949, Roche) and cholesterol (cat no. 03039773, Roche) were measured using the Cobas Integra 400 Plus (Roche), as per manufacturer’s instructions.

### Analysis of Gene Expression

RNA was extracted from snap-frozen LV using TRIzol^®^ reagent (Life Technologies), which was then DNAse treated (Life Technologies) and reverse-transcribed (Applied Biosystems), as per the manufacturer’s instructions. Relative LV expression was determined via real-time polymerase chain reaction using SYBR Green chemistry (Applied Biosystems), and primers generated from mouse sequences in GenBank (Supplementary Figure 2). Quantitative analysis was performed using the QuantStudio7 Flex system (Applied Biosystems), using the 2^–Δ^ ^Δ^ ^Ct^ method to detect fold differences relative to the defined comparison group.

### Histological Analyses

A portion of LV was fixed in 10% neutral buffered formalin overnight, embedded in paraffin and serially sectioned (4 μm sections for LV and liver, and 3 μm sections for kidney). LV sections were stained with picrosirius red (0.1% w/v) to assess cardiac interstitial collagen content (using polarised microscopy to differentiate type I [orange] and type III [green] collagen), or haemotoxylin and eosin (H&E), to quantify cardiomyocyte width. Sections were imaged (picrosirius red, x200 magnification; H&E, x100 magnification) using an Olympus BX43 microscope and quantified by digital image analysis in ImageJ. For the quantification, 10 cells (H&E) from 10 sections (H&E and picrosirius red) were analysed. Kidney sections were stained with periodic acid Schiff (PAS) for measurement of mesangial expansion as previously described (Watson et al., 2010). Sections were imaged (x400 magnification) using an Olympus BX43 microscope. For the quantification of the proportional area of staining, 15 glomeruli were analysed using Image-Pro Analyser 7.0 (Media Cybernetics). Liver sections were stained with haematoxylin and eosin as previously described (Ritchie et al., 2012). Scanned imaging was performed by the Monash University Histology Platform, blinded, and NAFLD activity scoring completed independently by pathologists at WuXi AppTec (Kleiner et al., 2005).

### Western Blotting

LV protein was extracted by homogenisation in ice-cold RIPA buffer and 60 μg loaded onto 10% SDS-PAGE gels before blotting on a polyvinylidene difluoride membrane (Immobilon-FL, Millipore). Membranes were probed overnight at 4°C with antibodies (1:1000) against CD36 (#ab133625, rabbit, Abcam), Bax (#2772, rabbit, Cell Signalling), β-Actin (#4967, rabbit, Cell Signalling), pJNK (#9251, rabbit, Cell Signalling) and total JNK (#9252, rabbit, Cell Signalling). This was followed by incubation with peroxidase-labelled anti-rabbit (#7074, goat, Cell Signalling) or anti-mouse (#7076, horse, Cell Signalling) secondary antibody (1:10,000) for 60 min at room temperature, before the membrane was imaged using the ChemiDoc imaging system (Bio-Rad), and quantified by densitometry using Image Lab software (Bio-Rad).

### Echocardiography

Echocardiography was performed in anaesthetised mice (ketamine/xylazine/atropine: 80/8/0.8 mg/kg i.p.) at study endpoint utilising a Philips iE33 ultrasound machine with 15 MHz linear (M-mode) and 12 MHz sector (Doppler and tissue Doppler) transducer. Echocardiography was carried out and analysis validated by the Baker Institute Echocardiography Platform. Technicians were blinded to treatment group. LV posterior wall (Pwd) thickness, LV chamber dimensions and fractional shortening were assessed from M-mode imaging. LV mass derived using following equation: (Awd + LVEDD + Pwd)^3^ – LVEDD^3^^∗^1.055. LV filling was assessed using transmitral Doppler flow; the ratio of early (E) and late (A) mitral flow velocities (E/A ratio) and E-wave deceleration time were measured. Tissue Doppler echocardiography was used to assess the ratio of e′ velocity and a′ velocity (e′/a′ ratio).

### Statistical Analysis

Data were analysed with GraphPad Prism 7.01 statistical software package. An unpaired *t*-test was performed to compare two groups. Two-way ANOVA was used to compare body weight, blood glucose and tolerance test data. Statistical significance was considered at *P* < 0.05.

## Results

### Characterisation of Diabetes and Organ Morphology

**FIGURE 1:**
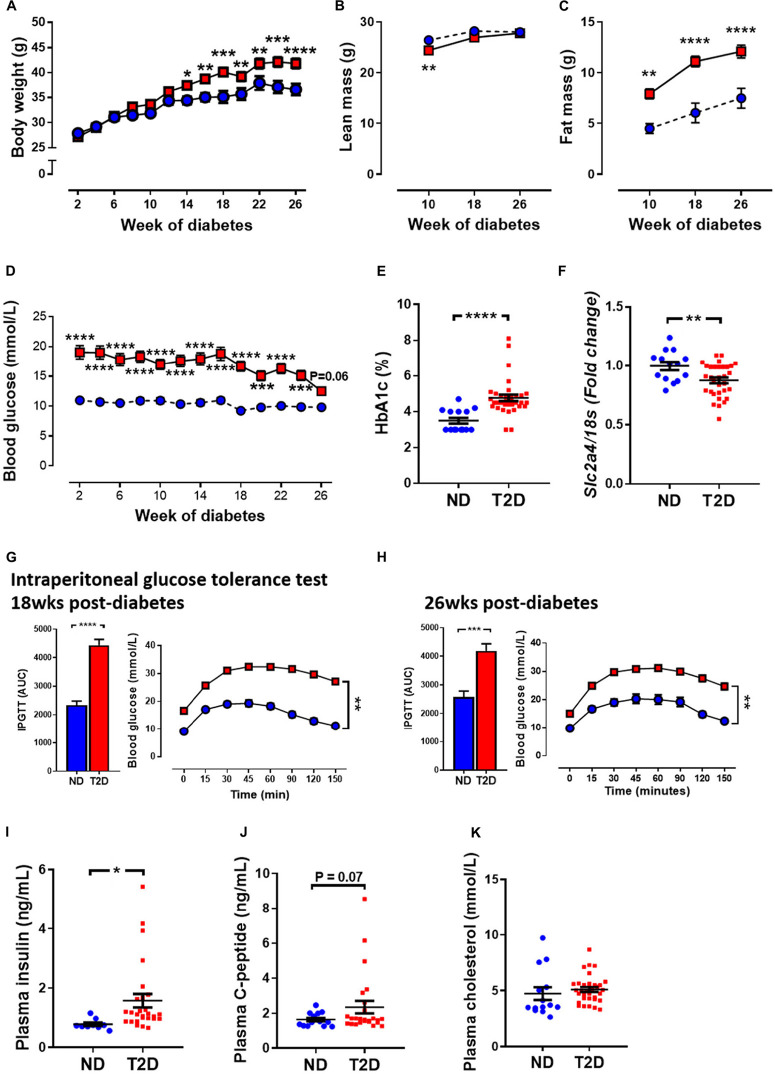
Effect of experimental diabetes (combining low-dose STZ and high fat diet) on metabolic characteristics. **(A)** Body weight, **(B)** lean mass, **(C)** fat mass, **(D)** blood glucose, **(E)** percentage glycated haemoglobin (HbA1c) and **(F)** LV *Slc2a4* gene expression (glucose transporter GLUT4). Intraperitoneal glucose tolerance test (IPGTT) at **(G)** 18 weeks and **(H)** 26 weeks. Plasma **(I)** insulin, **(J)** C-peptide levels and **(K)** cholesterol at 26 weeks. Data are presented as mean ± SEM. *n* = 9–33 per group (note individual data points). Data analysed using unpaired *t*-test. ^∗^*P* < 0.05, ^∗∗^*P* < 0.01, ^∗∗∗^*P* < 0.001, ^****^P < 0.0001 compared to ND. Blue circles ND; red squares T2DM. ND, non-diabetic; T2DM, type 2 diabetes; HbA1c, glycated haemoglobin; LV, left ventricle; STZ, streptozotocin, AUC, area-under-the-curve.

T2DM in patients is characterised by an increased in blood glucose, and often by an increase in fat mass, as well as changes in glucose handling. In this study, mice that received STZ and high-fat diet gained weight at a quicker rate than non-diabetic mice. This led to a significant increase in body weight at 14 weeks of diabetes and remained elevated until study ends (Figure 1A). Echo-MRI as a means to assess whole body composition in live subjects revealed that diabetic mice had lower absolute levels of lean mass at 10 weeks of diabetes, however there was no difference at 18 and 26 weeks of diabetes (Figure 1B). Fat mass in grams was elevated at all timepoints studied in diabetic mice (Figure 1C). Blood glucose levels were significantly elevated in diabetic mice at week 2 (ND 11.0 ± 0.44 vs. T2DM 19.1 ± 1.12, *P* < 0.0001), and these remained elevated throughout the study (Figure 1D). At study endpoint, glycated haemoglobin (HbA1c) levels, a long term measure of glucose control, was significantly elevated in diabetic mice (Figure 1E). In the heart, glucose transporter 4 (GLUT4) is the major transporter responsible for glucose uptake, however in diabetes there is a decrease in glucose utilisation and an increase in fatty acid consumption (Szablewski, 2017). LV mRNA levels of GLUT4 were significantly reduced in diabetic mice (Figure 1F).

Glucose tolerance test assesses glucose handling and is a common screen for T2DM in patients. T2DM mice exhibited impaired glucose tolerance as demonstrated by increased area-under-the-curve (AUC) by 18 weeks of diabetes (Figure 1G), which persisted to 26 weeks of diabetes (Figure 1H). T2DM mice also exhibited mild insulin resistance (Supplementary Figure 3). Furthermore, there was also a significant elevation in plasma insulin levels (Figure 1I) and a trend toward an elevation in C-peptide levels at 26 weeks in diabetic mice (Figure 1J). There was no difference in total cholesterol levels between the two cohorts (Figure 1K).

Tibia length, a marker of animal size was unchanged with diabetes (Table 1). Total heart weight and LV weight were also unchanged with diabetes, as were kidney and lung weights (Table 1). Diabetic mice exhibited significantly larger liver weight and spleen weight (Table 1), as well as increased weight of all fat depots measured: pericardial, perirenal and inguinal (Table 1).

**TABLE 1:** Organ weights at 26 weeks of diabetes.

	**26 weeks**	
	**ND**	**T2DM**
*N*	14	33
Tibial length (mm)	17.5 ± 0.1	17.4 ± 0.1
Heart weight (mg)	153 ± 3	159 ± 3
LV weight (mg)	113 ± 2	114 ± 2
Kidney weight (mg)	250 ± 5	241 ± 7
Lungs weight (mg)	164 ± 3	173 ± 6
Liver weight (g)	1.62 ± 0.1	2.18 ± 0.1^∗^
Spleen weight (mg)	105 ± 3	121 ± 5^∗^
Pericardial fat weight (mg)	21.9 ± 5.0	44.8 ± 3.8^∗^
Perirenal fat weight (mg)	732 ± 96	972 ± 63^∗^
Inguinal fat weight (mg)	553 ± 123	1210 ± 96^∗^

*Data are presented as mean ± SEM and analysed by unpaired *t*-test. ^∗^*P* < 0.05 vs. ND. LV, left ventricle; ND, non-diabetic; T2DM, type 2 diabetes.*

### Characterisation of Cardiac Function

**FIGURE 2:**
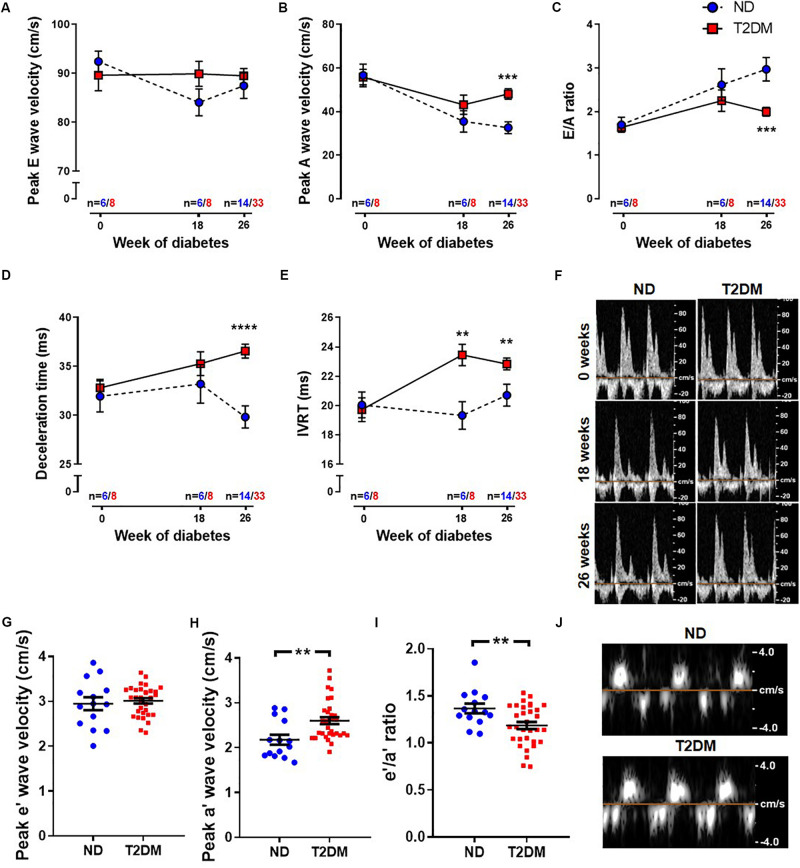
Effect of experimental diabetes on LV diastolic dysfunction. Quantification of **(A)** peak E wave velocity, **(B)** peak A wave velocity, **(C)** E/A ratio, **(D)** deceleration time and **(E)** IVRT obtained from pulsed-wave Doppler echocardiography at 0, 18 and 26 weeks of diabetes. **(F)** Representative mitral flow patterns from pulsed-wave Doppler echocardiography. Quantification of **(G)** peak e′ wave velocity, **(H)** peak a′ wave velocity and **(I)** e′/a′ ratio obtained from tissue Doppler echocardiography at 26 weeks of diabetes. **(J)** Representative images from tissue Doppler echocardiography. Data are presented as mean ± SEM. 0 and 18 weeks *n* = 6–8; 26 weeks *n* = 14–33 per group (note individual data points). Data analysed using unpaired *t*-test. ^∗^*P* < 0.05, ^∗∗^*P* < 0.01, ^∗∗∗^*P* < 0.001, ^****^P < 0.0001 compared to ND. Blue circles ND; red squares T2DM. ND, non-diabetic; T2DM, type 2 diabetes; IVRT, isovolumic relaxation time.

Patients with diabetes develop impairments in cardiac relaxation that if left untreated lead to heart failure, predominantly with preserved ejection fraction (Marwick et al., 2018). In order to asses cardiac function, echocardiography was carried out before the induction of diabetes (baseline, week 0), after 18 weeks of diabetes, and at study endpoint (26 weeks of diabetes). No differences in LV diastolic or systolic function were observed at baseline between experimental groups (Figure 2 and Table 1). After 18 weeks of diabetes, isovolumic relaxation time (IVRT), a marker of LV diastolic dysfunction, was increased in diabetic mice (Figure 2E). No other markers of LV diastolic function assessed were altered by diabetes at this timepoint. Notably, at 26 week of diabetes there was clear evidence of diastolic dysfunction, as assessed by Doppler and tissue Doppler echocardiography. There was no change in E wave velocity with diabetes (Figure 2A), however there was an increase in A wave velocity (Figure 2B) that led to a significant reduction in E/A ratio (Figure 2C). This was also corroborated by a prolongation in both deceleration time (Figure 2D) and IVRT (Figure 2E). Moreover, despite no change in e′ velocity (Figure 2G) there was an elevation in a′ velocity (Figure 2H), hence leading to a reduction in the e′/a′ ratio (Figure 2I). LV diastolic dysfunction is typically followed by systolic dysfunction, however, at these time points in this experimental model of diabetes, no differences in echocardiographic structure and systolic function were observed between groups, indicated by LV end-systolic/diastolic dimensions and fractional shortening (Table 2). It is important to note that heart rate also remained similar between the two groups of mice at the same age, for both LV diastolic and systolic measures (Table 2).

**TABLE 2:** Echocardiographic analysis of systolic heart function in anaesthetised ND and T2DM mice.

	**Baseline**		**18 weeks**		**26 weeks**	
	**ND**	**T2DM**	**ND**	**T2DM**	**ND**	**T2DM**
*n*	6	8	6	8	14	33
Heart rate (bpm)	459 ± 23	453 ± 17	375 ± 9	394 ± 16	364 ± 13	378 ± 7
Ex-LVEDD (mm)	5.11 ± 0.06	4.99 ± 0.11	5.70 ± 0.04	5.50 ± 0.05^∗^	5.49 ± 0.10	5.63 ± 0.03
AWd (mm)	0.76 ± 0.02	0.76 ± 0.02	0.74 ± 0.03	0.80 ± 0.02	0.77 ± 0.02	0.77 ± 0.01
LVEDD (mm)	3.66 ± 0.04	3.68 ± 0.04	4.12 ± 0.06	3.90 ± 0.04^∗∗^	4.18 ± 0.14	4.09 ± 0.04
PWd (mm)	0.75 ± 0.03	0.69 ± 0.02	0.77 ± 0.02	0.82 ± 0.02^∗^	0.77 ± 0.02	0.78 ± 0.02
LVESD (mm)	2.36 ± 0.07	2.32 ± 0.08	2.70 ± 0.03	2.56 ± 0.04^∗^	2.71 ± 0.05	2.70 ± 0.04
Fractional shortening (%)	35.7 ± 1.6	37.0 ± 1.7	35.3 ± 1.5	34.5 ± 0.8	34.4 ± 1.9	34.0 ± 0.6
Estimated LV mass (mg)	94 ± 4	89 ± 3	117 ± 2	115 ± 4	120 ± 7	117 ± 2
Estimated LV mass/BW (mg/g)					3.30 ± 0.14	2.84 ± 0.06^∗∗^
Estimated LV mass/TL (mg/mm)					6.87 ± 0.36	6.71 ± 0.10

*Data are presented as mean ± SEM and analysed by unpaired *t*-test. ^∗^*P* < 0.05, ^∗∗^*P* < 0.01 vs. ND. Ex-LVEDD, external LV end diastolic dimension; Awd, anterior wall diastolic thickness; LVEDD, LV end diastolic dimension; PWd, posterior wall diastolic thickness; LVESD, LV end systolic dimension; BW, body weight; TL, tibial length.*

### Characterisation of Cardiac Structural Changes

**FIGURE 3:**
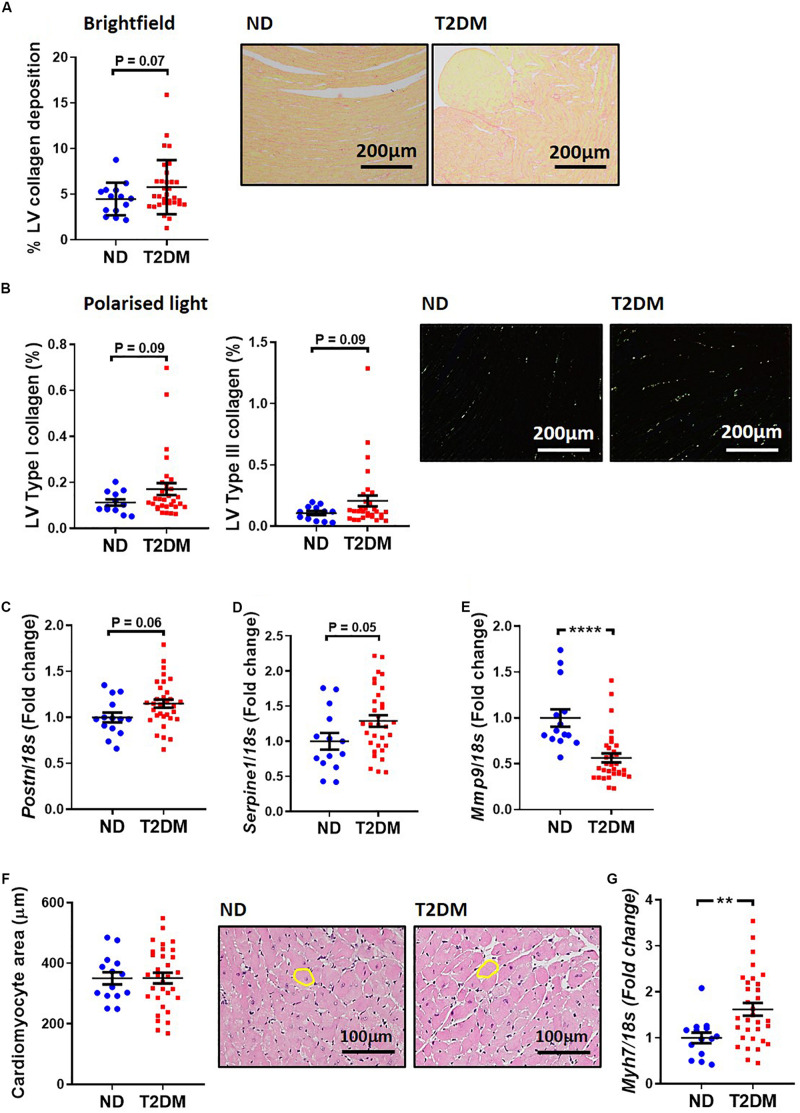
Effect of experimental diabetes on LV structural changes. **(A)** Quantification of total LV interstitial collagen in picrosirius red-stained sections; representative brightfield images shown (x200 magnification). **(B)** Quantification of type I and type III in picrosirius red-stained sections; representative polarised light images shown (x200 magnification). LV gene expression of markers of cardiac fibrosis; **(C)** *Postn* (periostin), **(D)** *Serpine1* (plasminogen activator inhibitor-1), **(E)** *Mmp9* (matrix metalloproteinase 9). **(F)** Quantification of total LV cardiomyocyte area in haemotoxylin and eosin-stained sections; representative brightfield images shown (x200 magnification). **(G)** LV gene expression of cardiomyocyte hypertrophy marker *Myh7* (β-myosin heavy chain). Data are presented as mean ± SEM. *n* = 12–31 per group (note individual data points). Data analysed using unpaired *t*-test. ^∗∗^*P* < 0.01, ^****^P < 0.0001 compared to ND. Blue circles ND; red squares T2DM. ND, non-diabetic; T2DM, type 2 diabetes; LV, left ventricle.

A common structural hallmark in the diabetic heart is increased fibrosis. In this experimental model of diabetes there was a tendency toward an increase in total LV interstitial collagen deposition, as analysed by picrosirius red staining (*P* = 0.07; Figure 3A). Polarised light was used to specifically quantify type I and type III collagen, and again there was a tendency for both types to be elevated between non-diabetic and diabetic mice (Figure 3B). Several markers of cardiac fibrosis were also analysed by real-time qPCR. mRNA levels of periostin (*P* = 0.06; Figure 3C) and plasminogen activator inhibitor 1 (*P* = 0.05; Figure 3D) tended to be increased in diabetic mice, whilst mRNA levels of matrix metallopeptidase 9, an extracellular matrix remodelling protein, was significantly reduced in diabetic mice (Figure 3E). Another hallmark associated with diabetic cardiomyopathy is cardiac and cardiomyocyte hypertrophy. In this model, there was no change in cardiomyocyte size (Figure 3F), however, there was a significant increase in β-myosin heavy chain gene expression, a marker of cardiac hypertrophy, in diabetic mice (Figure 3G).

### Effect of Diabetes on Cardiac Signalling Pathways

**FIGURE 4:**
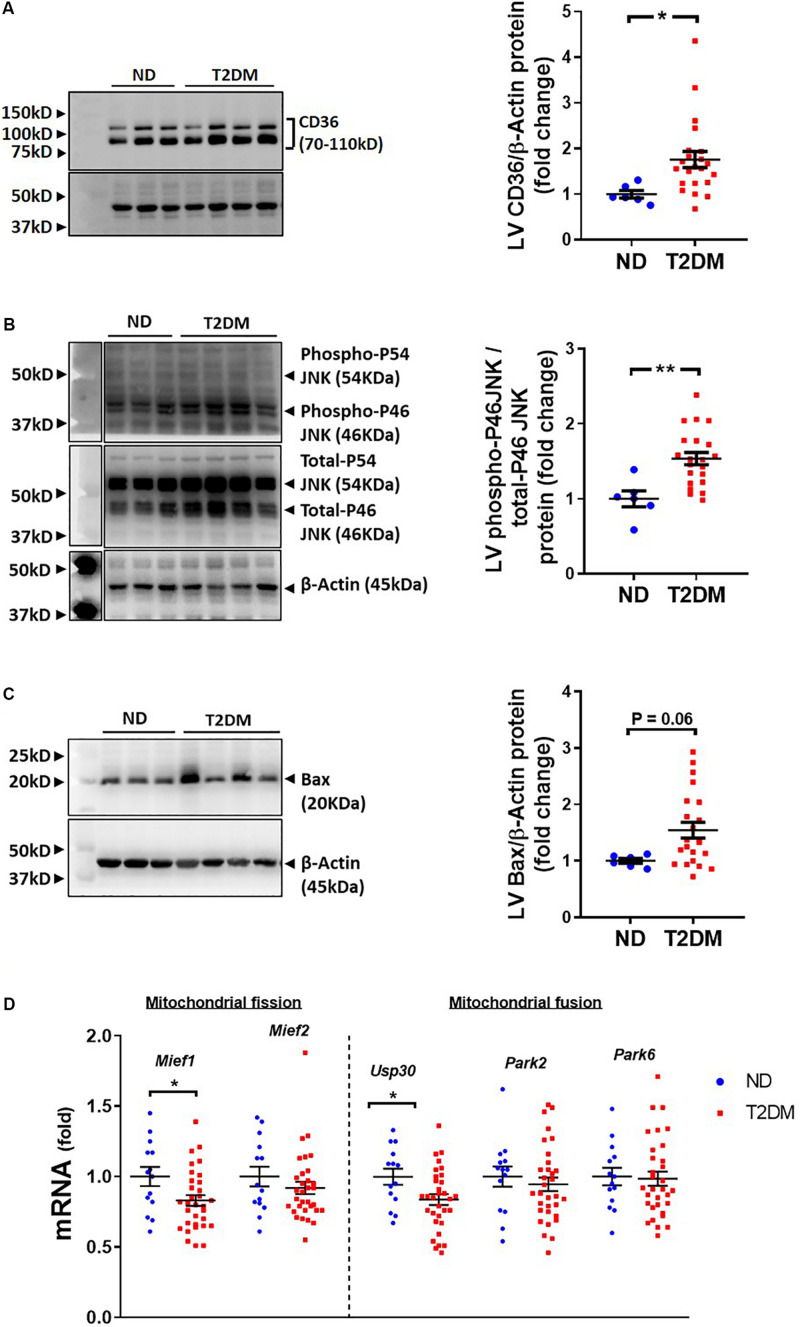
Effect of experimental diabetes on cardiac signalling pathways. Quantification data and representative images of western blot assessment of **(A)** CD36/β-Actin, **(B)** phospho-P46 JNK/total-P46 JNK and **(C)** Bax/β-Actin. Uncropped blots are included in Supplementary Figure 4. **(D)** LV gene expression of markers of mitochondrial fission and fusion; *Mief1*, *Mief2*, *Usp30*, *Park2* and *Park6*. Data are presented as mean ± SEM. *n* = 6–22 per group (note individual data points). Data analysed using unpaired *t*-test. ^∗^*P* < 0.05, ^∗∗^*P* < 0.01, ^∗∗∗^*P* < 0.001, ^****^P < 0.0001 compared to ND. Blue circles ND; red squares T2DM. ND, non-diabetic; T2DM, type 2 diabetes; LV, left ventricle.

Several pathophysiological mechanisms and cellular signalling pathways have been implicated in the development of diabetic cardiomyopathy (Bugger and Abel, 2008, 2009; Tate et al., 2017b). There was a significant increase in LV protein levels of fatty acid transport, CD36, in diabetic mice (Figure 4A). Cell injury markers JNK and Bax were assessed in mouse LV in this model of diabetes. The ratio of phosphorylated P46 JNK to total P46, an indication of the activated protein, was increased with diabetes (Figure 4B), whilst there was also a trend toward an increase in levels of Bax protein (*P* = 0.06; Figure 4C). LV expression of genes relating to mitochondrial fission and fusion were assessed. We observed that LV expression of *Mief1* and *USP30* were significantly reduced in diabetic mice (Figure 4D), although several other genes assessed (Mief2, Park2 and Park6) were not affected by the presence of diabetes (Figure 4D).

### Characterisation of Liver Structure and Function

**FIGURE 5:**
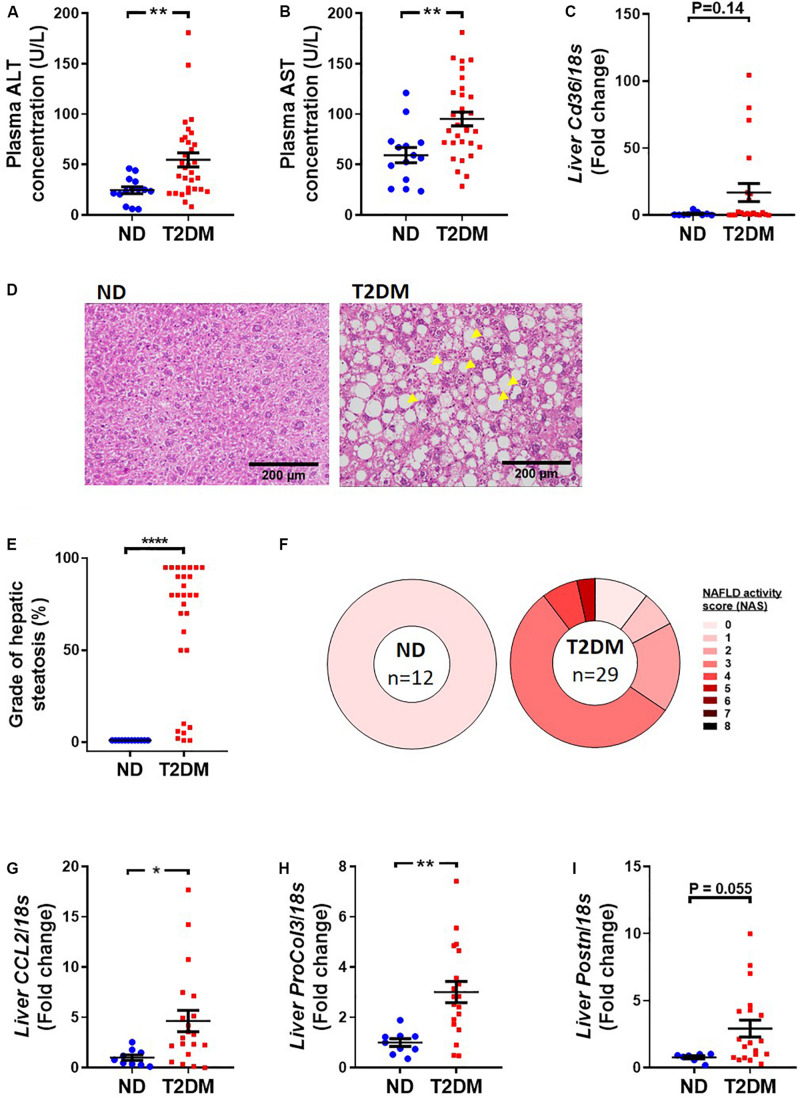
Effect of experimental diabetes on liver structure and function. Plasma **(A)** alanine aminotransferase (ALT) and **(B)** aspartate aminotransferase (AST) at 26 weeks. **(C)** Liver *Cd36* gene expression. **(D)** Representative images of liver haemotoxylin and eosin staining, **(E)** grade of steatosis and **(F)** NAFLD activity scoring. Liver **(G)** *Ccl2* (monocyte chemoattractant protein 1), **(H)** *ProCol3* (procollagen 3) and **(I)** *Postn* (periostin) gene expression. Data are presented as mean ± SEM. *n* = 8–33 per group (note individual data points). Data analysed using unpaired *t*-test. ^∗^*P* < 0.05, ^∗∗^*P* < 0.01, ^****^P < 0.0001 compared to ND. Blue circles ND; red squares T2DM. ND, non-diabetic; T2DM, type 2 diabetes; NAFLD, non-alcoholic fatty liver disease; NAS, NAFLD activity score.

Mice that received low-dose STZ and high-fat diet exhibited elevated levels of the liver enzymes, alanine aminotransferase (ALT; Figure 5A) and aspartate aminotransferase (AST; Figure 5B), as well as a trend toward an increase in *Cd36* mRNA levels (Figure 5C). Blinded pathological assessment of haemotoxylin and eosin (H&E; Figure 5D) stained liver sections revealed a marked increase in steatosis in diabetic mice (Figure 5E). There was some evidence of ballooning of hepatocytes in some diabetic mice but no noticeable difference in lobular inflammation (data not shown). NAFLD activity score was “0” in all non-diabetic mice, whilst the majority of diabetic mice had a NAFLD activity score of “3,” denoting “uncertain” NASH (Figure 5F). Real-time qPCR showed an increase in inflammatory marker CCL2 (Figure 5G), an increase in procollagen 3 (Figure 5H), and a trend toward an increase in periostin (*P* = 0.055; Figure 5I).

### Characterisation of Renal Structure and Function

**FIGURE 6:**
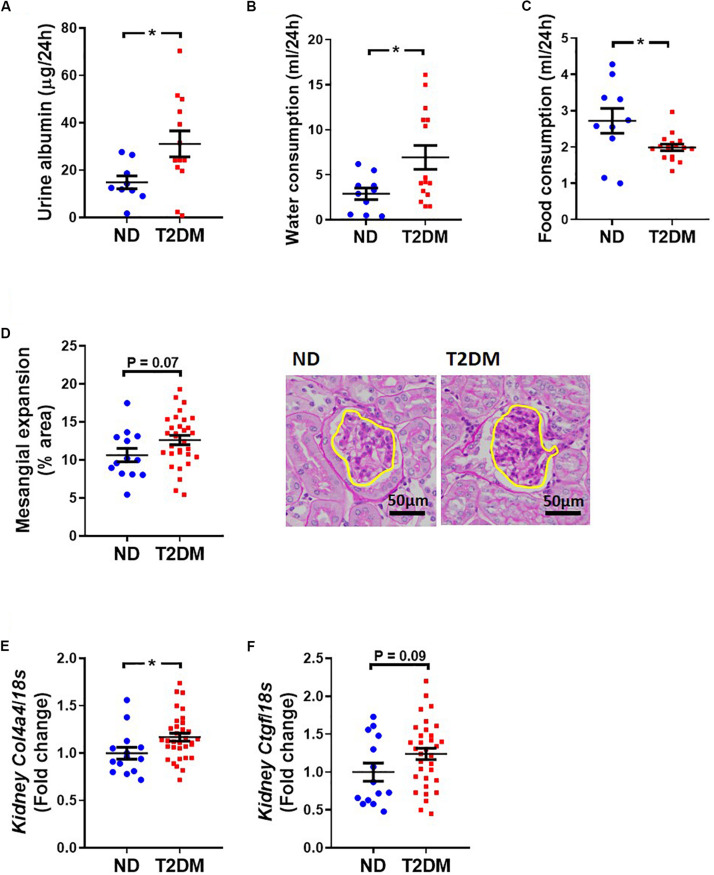
Effect on experimental diabetes on kidney structure and function. **(A)** Twenty four-hour urinary albumin excretion. Metabolic caging assessment of **(B)** food and **(C)** water consumption. **(D)** PAS stained glomeruli to assess mesangial expansion; mean data and representative images (x400 magnification). Kidney **(E)** *Col4a4* (collagen IV) and **(F)** *Ctgf* (CTGF) gene expression. Data are presented as mean ± SEM. *n* = 9–33 per group (note individual data points). Data analysed using unpaired *t*-test. ^∗^*P* < 0.05 compared to ND. Blue circles ND; red squares T2DM. ND, non-diabetic; T2DM, type 2 diabetes; CTGF, connective tissue growth factor; PAS, periodic acid–Schiff.

Albuminuria was significantly elevated in T2DM mice (Figure 6A). Metabolic caging revealed an elevation in water (Figure 6B) and a decrease in food (Figure 6C) consumption in diabetic mice. There was a trend toward an increase in kidney mesangial area (Figure 6D), however there was a significant increase in collagen IV gene expression (Figure 6E), and a tendency toward an increase in connective tissue growth factor gene expression (*P* = 0.09; Figure 6F).

## Discussion

This study reveals that a combination of low-dose STZ superimposed on high-fat diet over the longer-term mimics several features of T2DM, and most importantly, produces robust LV diastolic dysfunction at 26 weeks which is a characteristic of patients with T2DM-induced cardiomyopathy.

Many animal models of diabetes exist, each replicating certain aspects of clinical diabetes. These animal models take advantage of genetics, diets and pancreatic toxins to induce diabetes. In terms of diabetic cardiomyopathy, several models of diabetes have been shown to cause diastolic dysfunction, of which the STZ model of T1DM-induced cardiomyopathy is most commonly used. Several reviews have summarised common features of animal models of diabetic cardiomyopathy (Hsueh et al., 2007; Bugger and Abel, 2009), and the Diabetic Complications Consortium provides validation criteria for animal studies of diabetes-induced complications. More recently, several laboratories have devised protocols that use a dietary intervention superimposed on the pancreatic toxin, STZ, to induce T2DM (Barrière et al., 2018; Wanrooy et al., 2018). This model exhibits diastolic dysfunction comparable to the *db/db* mouse but with a milder systemic phenotype, more closely resembling the progression of systemic metabolic changes observed in humans with T2DM, including progressive gains in body weight and fat mass. Furthermore, whereas genetic models with impairments in leptin signalling begin at birth, this combination approach was implemented from 6 weeks of age. The combination strategy is necessary as dietary intervention alone, a major driver of diabetes in the human population, only replicates the clinical features of prediabetes or early-stage T2DM, whereas addition of the pancreatic toxin elicits β-cell failure which is a common feature of later-stage diabetes (Reed et al., 2000; Lee et al., 2015). A further advantage of this method is that it is amenable to be superimposed on genetic mouse models.

In this study, we followed the mice for 26 weeks from the induction of diabetes. In STZ models of T1DM-induced cardiomyopathy, cardiac dysfunction is evident after 8 weeks of diabetes (Huynh et al., 2010; Prakoso et al., 2017). However, 18 and 26 weeks of diabetes were the chosen time points in the current study as this combination strategy is less severe, meaning complications would likely take longer to develop. In another study using a combination of dietary intervention and the pancreatic β-cell toxin STZ in a rat, disease progression appears to fall into several phases, mimicking the clinical situation. Indeed, in this recent study from Barrière et al. (2018) utilising a high fat, high fructose and low-dose STZ regime described three distinct phases of disease progression. The first phase, or prediabetic phase, was characterised by hyperinsulinaemia and hyperglycaemia with no signs of obesity. Our study, albeit using a slightly different protocol, corroborated these findings, with hyperglycaemia present at 10 weeks after induction, with no change in total body weight. Notably, although there was no change in total body weight, body composition analysis revealed a reduction in lean mass at 10 weeks in diabetic mice, but a significant increase in absolute fat mass and percentage fat mass. In the latter stages of disease progression, Barrière et al. (2018) describe normalisation of blood glucose, polydipsia and polyuria, findings replicated in our study. In this study, after 26 weeks of diabetes an increase in glycated haemoglobin, insulin and C-peptide was also present.

Cardiovascular complications are the leading cause of death in patients with T2DM, accounting for up to 80% of mortality in this group (Gaede et al., 2008). In fact, both cardiac stiffness and subclinical diastolic dysfunction can be detected in approximately 60% of optimally-treated patients. The diabetic heart is prone to progressive degenerative changes which have limited capacity for repair (Tate et al., 2017b). A notable feature of the diabetic heart, in contrast to other forms of cardiac function, is a susceptibility to extracellular matrix remodelling, including myocardial fibrosis, cardiomyocyte hypertrophy and apoptosis, resulting in deficiencies in myocardial relaxation (Tate et al., 2017b). As such, the primary aim of this study was to investigate the suitability of the combination of a high fat and low dose STZ as a non-genetic model of type 2 diabetes to study diabetic cardiomyopathy. This is necessary as the underlying mechanisms of diabetic cardiomyopathy are not fully understood partly due to the lack of a suitable animal model, but also because of the difficulty of differentiating between the direct insult of diabetes on the heart versus the indirect contribution of other cardiovascular risk factors (Riehle and Abel, 2016; Tate et al., 2017b).

LV diastolic dysfunction with preserved ejection fraction is often the first detectable functional change in the progression of diabetic cardiomyopathy in humans, as observed in several clinical studies (Brunvand et al., 2016). In the current study, 18 weeks after the induction of diabetes there was some evidence of the development of diastolic dysfunction in diabetic mice compared to their non-diabetic counterparts. Moreover, at 26 weeks of diabetes, LV diastolic dysfunction had clearly manifest, illustrated by changes in several markers from both Doppler and tissue Doppler echocardiography. These findings are consistent with other pre-clinical models of both T1DM and T2DM-induced cardiomyopathy (Prakoso et al., 2017; Tate et al., 2017a). In patients, early diastolic dysfunction is often succeeded by LV systolic dysfunction, or by heart failure with reduced ejection fraction (HFrEF) (Fang et al., 2005; Tate et al., 2017b). However, conflicting reports have also described preserved, or even augmented systolic function (Romanens et al., 1999; Schannwell et al., 2002). In the current study, we did not observe any significant changes in LV systolic function, although extending the length of diabetes may be necessary to observe these changes and hence make a definitive conclusion (Table 1).

This animal model successfully replicated one prominent feature of human clinical diabetic cardiomyopathy, that being the presence of myocardial fibrosis. Indeed, interstitial type I and type III collagen tended to be elevated in diabetic mice in this study. Other experimental studies have described increased levels of replacement myocardial fibrosis in both T1DM and T2DM models, both in the interstitial and perivascular regions (Mizushige et al., 2000; Singh et al., 2008; Prakoso et al., 2017; Tate et al., 2017a). In patients, elevated collagen deposition has been reported in myocardial biopsies from T2DM-induced cardiomyopathy patients, i.e., absent of concomitant cardiovascular risk factors such as hypertension and atherosclerosis (Shimizu et al., 1993). Furthermore, markers of myocardial fibrosis have been correlated with the presence of diastolic dysfunction (Ihm et al., 2007). In regards to a potential mechanism, several growth factors including TGF-β and CTGF have been implicated in extracellular matrix remodelling, in particular the increase in myocardial fibrosis. In the current study, we observed an increase in periostin, a protein known to interact with extracellular matrix proteins including fibronectin and matrix metalloproteinases (MMPs) (Norris et al., 2007; Kii et al., 2010). Previously, a decrease in expression of MMP-2 was shown to attenuate extracellular matrix degradation (van Linthout et al., 2008). Whilst in an aged MMP-9 knockout mouse model, periostin had a role in regulating myocardial turnover and deposition (Chiao et al., 2012). Notably in the current study, we observed a decrease in the expression of MMP-9.

Although LV hypertrophy is a common feature of diabetic cardiomyopathy, there was no change in LV mass in the current study (Boudina and Abel, 2007). We also assessed the impact of diabetes on cardiomyocyte hypertrophy, another common feature in human and animal models of diabetes-induced heart failure (Tate et al., 2017b; Marwick et al., 2018). In the current study there was no change in cardiomyocyte size, as assessed using H&E staining, however there was an increase in β-MHC gene expression, a pathological marker of cardiomyocyte hypertrophy. That said, the structural phenotype of cardiomyocytes in diabetic cardiomyopathy is less clear, with opposing data reported in the literature in regards to cardiomyotcyte size (Frustaci et al., 2000; Di Bonito et al., 2005), or even a disorganised phenotype with both hypertrophic and atrophic cardiomyocytes (van Hoeven and Factor, 1990).

One common complication of T2DM is diabetic nephropathy (Retnakaran et al., 2006). Glomerular injury occurs in diabetic nephropathy and is characterised by thickening of the glomerular basement membrane, mesangial matrix expansion and an increase in glomerular permeability (De Blasio et al., 2017). Urinary albumin excretion measurements revealed an increase in diabetic mice, a common finding in several other models of diabetes (Watson et al., 2010). Furthermore, in our study we observed an increase in glomerular mesangial expansion which is known to be a driver of tubulointerstitial fibrosis (Katz et al., 2002; Shi et al., 2015). Indeed, several experimental models of diabetes, including from our own laboratory in STZ-induced T1DM reported an increase in collagen content in the glomerular of diabetic kidneys (De Blasio et al., 2017; Barrière et al., 2018), a feature replicated in this study observed as an increase in renal collagen IV mRNA. The molecular mechanisms driving these pathological changes are reasonably well-understood, including the upregulation of pro-sclerotic growth factors including CTGF and TGF-β (Lassila et al., 2005; Watson et al., 2010; Lan, 2011). Indeed, in the current study we report a non-statistically significant increase in CTGF at the mRNA level.

### Study Limitations

Only male mice were included in the current study, which is a clear limitation. This is important when cardiovascular risk is greater in women with diabetes than men (Kannel et al., 1974). A previous study reported exaggerated diastolic dysfunction, albeit with increased variability, and less pronounced hyperglycaemia in STZ-induced female mice, compared to male (Chandramouli et al., 2018). For these reasons, only male mice were included in the current study: (1) to increase likelihood of observing hyperglycaemia (which is less marked in this T2DM model); (2) to reduce the number of mice required, given the increased variability in cardiac functional measurements in previous studies. Future studies will include female mice. Wanrooy et al. (2018) recently compared the metabolic phenotype of control mice, STZ-induced mice, high fat diet-fed mice and the combination approach. Although Wanrooy et al. (2018) did not assess cardiac function, it would be of interest to compare the relative contribution of each treatment in relation to cardiac functional and structural changes. Furthermore, although beyond the scope of the current project, a more detailed characterisation of mitochondrial energetics and calcium handling in this model would be of particular interest. This model did not exhibit an increase in cardiomyocyte size in diabetic mice at the time point assessed, despite there being a significant elevation in β-myosin heavy chain gene expression, a marker of cardiac hypertrophy. Although cardiac dysfunction is present at this time point, assessing cardiomyocyte size after a longer period of diabetes would be of interest. It would also be interesting to assess cardiac fibrosis after a longer period of diabetes. Assessing MMP9 protein activity, in addition to gene expression analysis, would help understand the interplay between extracellular matrix turnover and cardiac fibrosis.

Taken together, our data illustrate that a combination of low-dose STZ and high-fat diet emulate several important metabolic changes seen in T2DM, as well as the development of diastolic dysfunction with preserved systolic function. This potentially provides a better T2DM mouse model to study cardiac dysfunction that mimics several facets observed in the clinic. This is particularly important as the current treatment strategies to treat heart failure in diabetic and non-diabetics are the same, despite the pathophysiological mechanisms underlying disease progression being distinct.

## Data Availability Statement

The datasets generated for this study are available on request to the corresponding author.

## Ethics Statement

The animal study was reviewed and approved by Alfred Medical Research Education Precinct Animal Ethics Committee.

## Author Contributions

MT, DP, MJD, and RR conceived and designed the research, drafted the manuscript, and edited and revised the manuscript. MT, DP, AMW, CP, MD, CQ, JW, CC, DN, AR, AS, HK, DD, AMDW, MJD, and RR performed the experiments. MT, DP, AMW, CP, MD, CQ, JW, CC, DN, AR, HK, DD, AMDW, MJD, and RR analyzed the data. MT, DP, CP, DN, CQ, MJD, and RR prepared the figures. MT, DP, AMW, MD, JW, CC, DN, AR, HK, DD, JD, AMDW, MJD, and RR approved final version of the manuscript.

## Conflict of Interest

The authors declare that the research was conducted in the absence of any commercial or financial relationships that could be construed as a potential conflict of interest.

## Supplementary Material

FIGURE S1 Flow chart of animal use based on the CONSAERT template for preclinical studies. ND, non-diabetic; T2D, diabetic.

FIGURE S2 Primer sequences for quantitative real-time PCR.

FIGURE S3 Intraperitoneal insulin tolerance test at 18wks and 26 wks post-diabetes.

FIGURE S4 Uncropped immunoblot for CD36, JNK, BAX and beta-actin. Red boxes depict cropped area included in the main manuscript.

## Abbreviations

AUC: area-under-the-curve
Awd: anterior wall diastolic thickness
BW: body weight
Ex-LVEDD: external LV end diastolic dimension
GLUT4: glucose transporter 4
HbA1c: glycated haemoglobin
HFpEF: heart failure with preserved ejection fraction
HFrEF: heart failure with reduced ejection fraction
IPGTT: intraperitoneal glucose tolerance test
IVRT: isovolumic relaxation time
LV: left ventricle
LVEDD: LV end diastolic dimension
LVESD: LV end systolic dimension
NAFLD: non-alcoholic fatty liver disease
NAS: NAFLD activity score
ND: non-diabetic
PAS: periodic acid–Schiff
Pwd: posterior wall dimension
SEM: standard error of the mean
STZ: streptozotocin
T1DM: type 1 diabetes
T2DM: type 2 diabetes
TL: tibial length.
