# Corrigendum: Characterising an Alternative Murine Model of Diabetic Cardiomyopathy

**DOI:** 10.3389/fphys.2021.734320

**Published:** 2021-08-19

**Authors:** Mitchel Tate, Darnel Prakoso, Andrew M. Willis, Cheng Peng, Minh Deo, Cheng Xue Qin, Jesse L. Walsh, David M. Nash, Charles D. Cohen, Alex K. Rofe, Arpeeta Sharma, Helen Kiriazis, Daniel G. Donner, Judy B. De Haan, Anna M. D. Watson, Miles J. De Blasio, Rebecca H. Ritchie

**Affiliations:** ^1^Heart Failure Pharmacology, Baker Heart and Diabetes Institute, Melbourne, VIC, Australia; ^2^Department of Diabetes, Central Clinical School, Monash University, Melbourne, VIC, Australia; ^3^School of Biosciences, The University of Melbourne, Melbourne, VIC, Australia; ^4^Oxidative Stress Laboratory, Baker Heart and Diabetes Institute, Melbourne, VIC, Australia; ^5^Preclinical Cardiology, Microsurgery and Imaging Platform, Baker Heart and Diabetes Institute, Melbourne, VIC, Australia; ^6^Department of Pharmacology and Therapeutics, The University of Melbourne, Melbourne, VIC, Australia

**Keywords:** diabetes, type 2 diabetes, diabetic cardiomyopathy, cardiac, experimental model

In the original article, there was a mistake in Figures 1G and H as published. We have noticed that the Y-axis units of both bar graphs are incorrect. The data in these figures is based on the area under the curve (AUC) measurement from glucose tolerance test graphs. We have noticed that the Y-values for glucose have not been multiplied by the time on the X-axis, but rather the row number in the spreadsheet in Prism. The time values had been pasted into the title column and not the x-values column. By correcting the graphs, the actual outcomes for these graphs does not change the results or outcomes of the manuscript. The corrected Figures 1G and H and the entire Figure 1 appears below.

**Figure 1:**
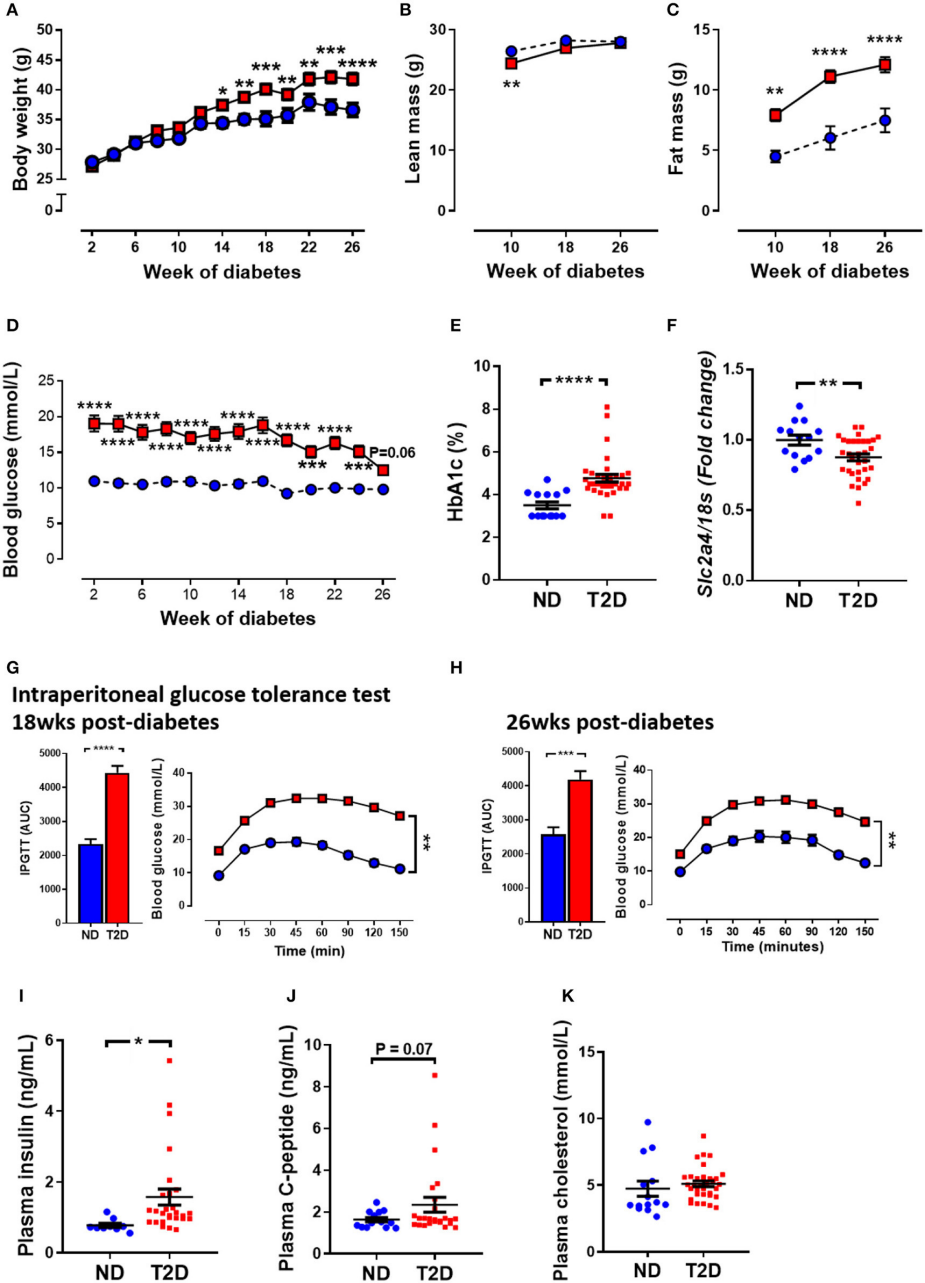
Effect of experimental diabetes (combining low-dose STZ and high fat diet) on metabolic characteristics. **(A)** Body weight, **(B)** lean mass, **(C)** fat mass, **(D)** blood glucose, **(E)** percentage glycated haemoglobin (HbA1c) and **(F)** LV *Slc2a4* gene expression (glucose transporter GLUT4). Intraperitoneal glucose tolerance test (IPGTT) at **(G)** 18 weeks and **(H)** 26 weeks. Plasma **(I)** insulin, **(J)** C-peptide levels and **(K)** cholesterol at 26 weeks. Data are presented as mean ± SEM. *n* = 9–33 per group (note individual data points). Data analysed using unpaired *t*-test. ^*^p < 0.05, ^**^p < 0.01, ^***^p < 0.001, ^****^p < 0.0001 compared to ND. Blue circles ND; red squares T2DM. ND, non-diabetic; T2DM, type 2 diabetes; HbA1c, glycated haemoglobin; LV, left ventricle; STZ, streptozotocin, AUC, area-under-the-curve.

The authors apologise for this error and state that this does not change the scientific conclusions of the article in any way. The original article has been updated.

