# Supplementary material for: Characterising an Alternative Murine Model of Diabetic Cardiomyopathy
Source: Front Physiol. 2019 Nov 14;10:1395. doi: 10.3389/fphys.2019.01395 (PMC6868003; doi:10.3389/fphys.2019.01395)
Supplement: FIGURE S1 — Flow chart of animal use based on the CONSAERT template for preclinical studies. ND, non-diabetic; T2D, diabetic. [file Presentation_1.PPTX]

## Slide 1
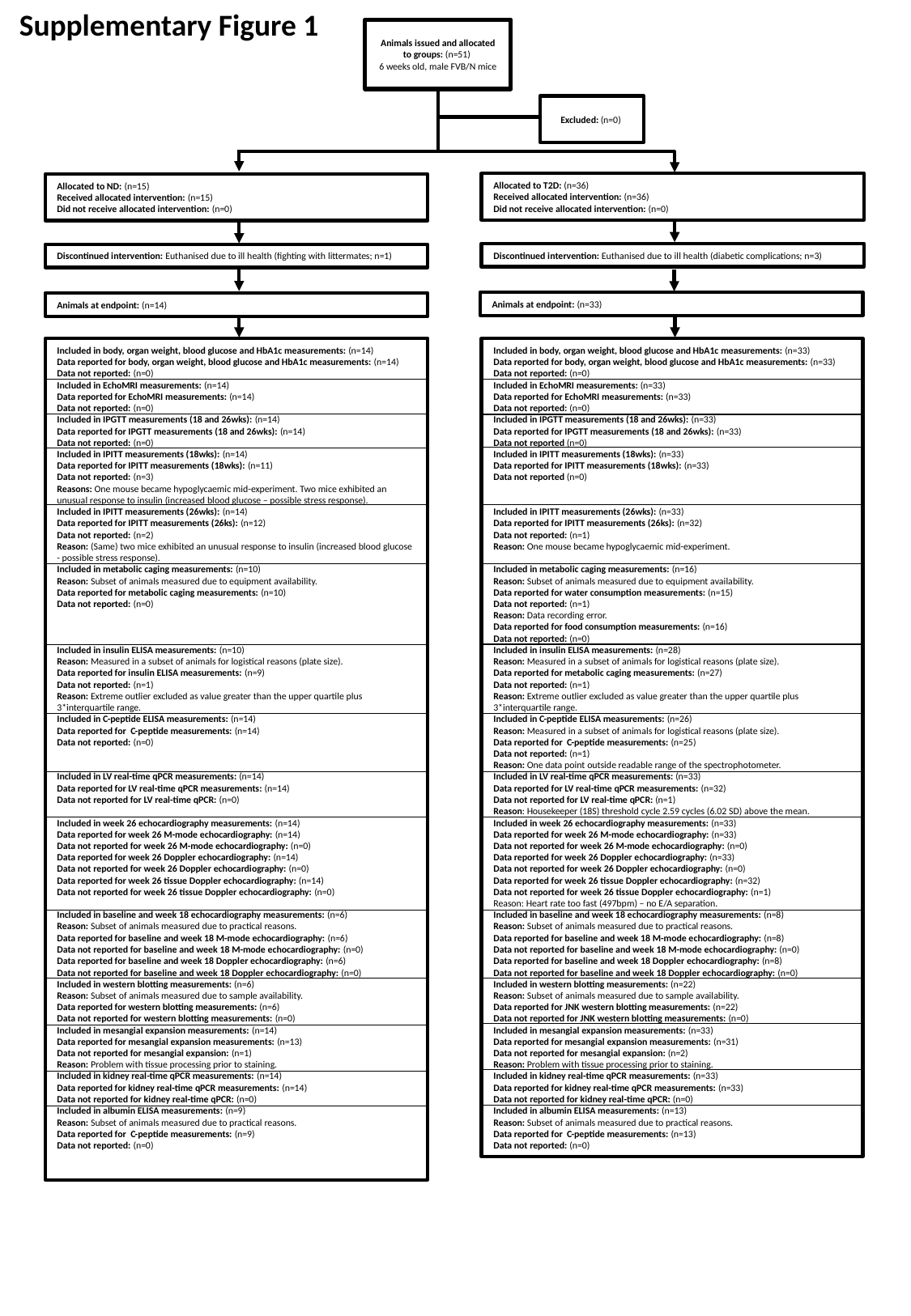

Supplementary Figure 1
Animals issued and allocated to groups: (n=51)
6 weeks old, male FVB/N mice
Excluded: (n=0)
Allocated to T2D: (n=36)
Received allocated intervention: (n=36)
Did not receive allocated intervention: (n=0)
Allocated to ND: (n=15)
Received allocated intervention: (n=15)
Did not receive allocated intervention: (n=0)
Discontinued intervention: Euthanised due to ill health (diabetic complications; n=3)
Discontinued intervention: Euthanised due to ill health (fighting with littermates; n=1)
Animals at endpoint: (n=33)
Animals at endpoint: (n=14)
Included in body, organ weight, blood glucose and HbA1c measurements: (n=14)
Data reported for body, organ weight, blood glucose and HbA1c measurements: (n=14)
Data not reported: (n=0)
Included in EchoMRI measurements: (n=14)
Data reported for EchoMRI measurements: (n=14)
Data not reported: (n=0)
Included in IPGTT measurements (18 and 26wks): (n=14)
Data reported for IPGTT measurements (18 and 26wks): (n=14)
Data not reported: (n=0)
Included in IPITT measurements (18wks): (n=14)
Data reported for IPITT measurements (18wks): (n=11)
Data not reported: (n=3)
Reasons: One mouse became hypoglycaemic mid-experiment. Two mice exhibited an unusual response to insulin (increased blood glucose – possible stress response).
Included in IPITT measurements (26wks): (n=14)
Data reported for IPITT measurements (26ks): (n=12)
Data not reported: (n=2)
Reason: (Same) two mice exhibited an unusual response to insulin (increased blood glucose - possible stress response).
Included in metabolic caging measurements: (n=10)
Reason: Subset of animals measured due to equipment availability.
Data reported for metabolic caging measurements: (n=10)
Data not reported: (n=0)
Included in insulin ELISA measurements: (n=10)
Reason: Measured in a subset of animals for logistical reasons (plate size).
Data reported for insulin ELISA measurements: (n=9)
Data not reported: (n=1)
Reason: Extreme outlier excluded as value greater than the upper quartile plus 3*interquartile range.
Included in C-peptide ELISA measurements: (n=14)
Data reported for C-peptide measurements: (n=14)
Data not reported: (n=0)
Included in LV real-time qPCR measurements: (n=14)
Data reported for LV real-time qPCR measurements: (n=14)
Data not reported for LV real-time qPCR: (n=0)
Included in week 26 echocardiography measurements: (n=14)
Data reported for week 26 M-mode echocardiography: (n=14)
Data not reported for week 26 M-mode echocardiography: (n=0)
Data reported for week 26 Doppler echocardiography: (n=14)
Data not reported for week 26 Doppler echocardiography: (n=0)
Data reported for week 26 tissue Doppler echocardiography: (n=14)
Data not reported for week 26 tissue Doppler echocardiography: (n=0)
Included in baseline and week 18 echocardiography measurements: (n=6)
Reason: Subset of animals measured due to practical reasons.
Data reported for baseline and week 18 M-mode echocardiography: (n=6)
Data not reported for baseline and week 18 M-mode echocardiography: (n=0)
Data reported for baseline and week 18 Doppler echocardiography: (n=6)
Data not reported for baseline and week 18 Doppler echocardiography: (n=0)
Included in western blotting measurements: (n=6)
Reason: Subset of animals measured due to sample availability.
Data reported for western blotting measurements: (n=6)
Data not reported for western blotting measurements: (n=0)
Included in mesangial expansion measurements: (n=14)
Data reported for mesangial expansion measurements: (n=13)
Data not reported for mesangial expansion: (n=1)
Reason: Problem with tissue processing prior to staining.
Included in kidney real-time qPCR measurements: (n=14)
Data reported for kidney real-time qPCR measurements: (n=14)
Data not reported for kidney real-time qPCR: (n=0)
Included in albumin ELISA measurements: (n=9)
Reason: Subset of animals measured due to practical reasons.
Data reported for C-peptide measurements: (n=9)
Data not reported: (n=0)
Included in body, organ weight, blood glucose and HbA1c measurements: (n=33)
Data reported for body, organ weight, blood glucose and HbA1c measurements: (n=33)
Data not reported: (n=0)
Included in EchoMRI measurements: (n=33)
Data reported for EchoMRI measurements: (n=33)
Data not reported: (n=0)
Included in IPGTT measurements (18 and 26wks): (n=33)
Data reported for IPGTT measurements (18 and 26wks): (n=33)
Data not reported (n=0)
Included in IPITT measurements (18wks): (n=33)
Data reported for IPITT measurements (18wks): (n=33)
Data not reported (n=0)
Included in IPITT measurements (26wks): (n=33)
Data reported for IPITT measurements (26ks): (n=32)
Data not reported: (n=1)
Reason: One mouse became hypoglycaemic mid-experiment.
Included in metabolic caging measurements: (n=16)
Reason: Subset of animals measured due to equipment availability.
Data reported for water consumption measurements: (n=15)
Data not reported: (n=1)
Reason: Data recording error.
Data reported for food consumption measurements: (n=16)
Data not reported: (n=0)
Included in insulin ELISA measurements: (n=28)
Reason: Measured in a subset of animals for logistical reasons (plate size).
Data reported for metabolic caging measurements: (n=27)
Data not reported: (n=1)
Reason: Extreme outlier excluded as value greater than the upper quartile plus 3*interquartile range.
Included in C-peptide ELISA measurements: (n=26)
Reason: Measured in a subset of animals for logistical reasons (plate size).
Data reported for C-peptide measurements: (n=25)
Data not reported: (n=1)
Reason: One data point outside readable range of the spectrophotometer.
Included in LV real-time qPCR measurements: (n=33)
Data reported for LV real-time qPCR measurements: (n=32)
Data not reported for LV real-time qPCR: (n=1)
Reason: Housekeeper (18S) threshold cycle 2.59 cycles (6.02 SD) above the mean.
Included in week 26 echocardiography measurements: (n=33)
Data reported for week 26 M-mode echocardiography: (n=33)
Data not reported for week 26 M-mode echocardiography: (n=0)
Data reported for week 26 Doppler echocardiography: (n=33)
Data not reported for week 26 Doppler echocardiography: (n=0)
Data reported for week 26 tissue Doppler echocardiography: (n=32)
Data not reported for week 26 tissue Doppler echocardiography: (n=1)
Reason: Heart rate too fast (497bpm) – no E/A separation.
Included in baseline and week 18 echocardiography measurements: (n=8)
Reason: Subset of animals measured due to practical reasons.
Data reported for baseline and week 18 M-mode echocardiography: (n=8)
Data not reported for baseline and week 18 M-mode echocardiography: (n=0)
Data reported for baseline and week 18 Doppler echocardiography: (n=8)
Data not reported for baseline and week 18 Doppler echocardiography: (n=0)
Included in western blotting measurements: (n=22)
Reason: Subset of animals measured due to sample availability.
Data reported for JNK western blotting measurements: (n=22)
Data not reported for JNK western blotting measurements: (n=0)
Included in mesangial expansion measurements: (n=33)
Data reported for mesangial expansion measurements: (n=31)
Data not reported for mesangial expansion: (n=2)
Reason: Problem with tissue processing prior to staining.
Included in kidney real-time qPCR measurements: (n=33)
Data reported for kidney real-time qPCR measurements: (n=33)
Data not reported for kidney real-time qPCR: (n=0)
Included in albumin ELISA measurements: (n=13)
Reason: Subset of animals measured due to practical reasons.
Data reported for C-peptide measurements: (n=13)
Data not reported: (n=0)

## Slide 2
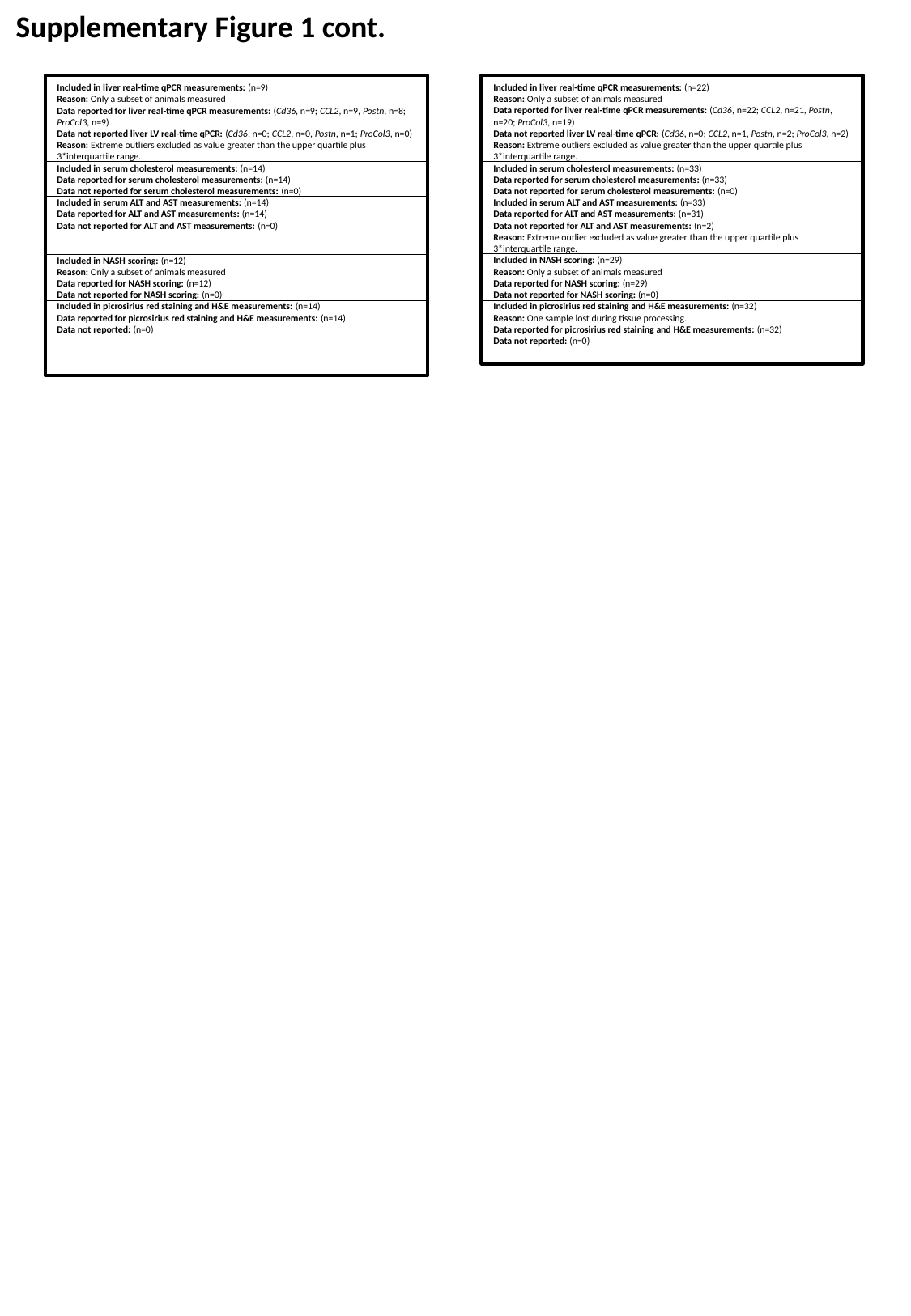

Supplementary Figure 1 cont.
Included in liver real-time qPCR measurements: (n=9)
Reason: Only a subset of animals measured
Data reported for liver real-time qPCR measurements: (Cd36, n=9; CCL2, n=9, Postn, n=8; ProCol3, n=9)
Data not reported liver LV real-time qPCR: (Cd36, n=0; CCL2, n=0, Postn, n=1; ProCol3, n=0)
Reason: Extreme outliers excluded as value greater than the upper quartile plus 3*interquartile range.
Included in serum cholesterol measurements: (n=14)
Data reported for serum cholesterol measurements: (n=14)
Data not reported for serum cholesterol measurements: (n=0)
Included in serum ALT and AST measurements: (n=14)
Data reported for ALT and AST measurements: (n=14)
Data not reported for ALT and AST measurements: (n=0)
Included in NASH scoring: (n=12)
Reason: Only a subset of animals measured
Data reported for NASH scoring: (n=12)
Data not reported for NASH scoring: (n=0)
Included in picrosirius red staining and H&E measurements: (n=14)
Data reported for picrosirius red staining and H&E measurements: (n=14)
Data not reported: (n=0)
Included in liver real-time qPCR measurements: (n=22)
Reason: Only a subset of animals measured
Data reported for liver real-time qPCR measurements: (Cd36, n=22; CCL2, n=21, Postn, n=20; ProCol3, n=19)
Data not reported liver LV real-time qPCR: (Cd36, n=0; CCL2, n=1, Postn, n=2; ProCol3, n=2)
Reason: Extreme outliers excluded as value greater than the upper quartile plus 3*interquartile range.
Included in serum cholesterol measurements: (n=33)
Data reported for serum cholesterol measurements: (n=33)
Data not reported for serum cholesterol measurements: (n=0)
Included in serum ALT and AST measurements: (n=33)
Data reported for ALT and AST measurements: (n=31)
Data not reported for ALT and AST measurements: (n=2)
Reason: Extreme outlier excluded as value greater than the upper quartile plus 3*interquartile range.
Included in NASH scoring: (n=29)
Reason: Only a subset of animals measured
Data reported for NASH scoring: (n=29)
Data not reported for NASH scoring: (n=0)
Included in picrosirius red staining and H&E measurements: (n=32)
Reason: One sample lost during tissue processing.
Data reported for picrosirius red staining and H&E measurements: (n=32)
Data not reported: (n=0)

## Slide 3
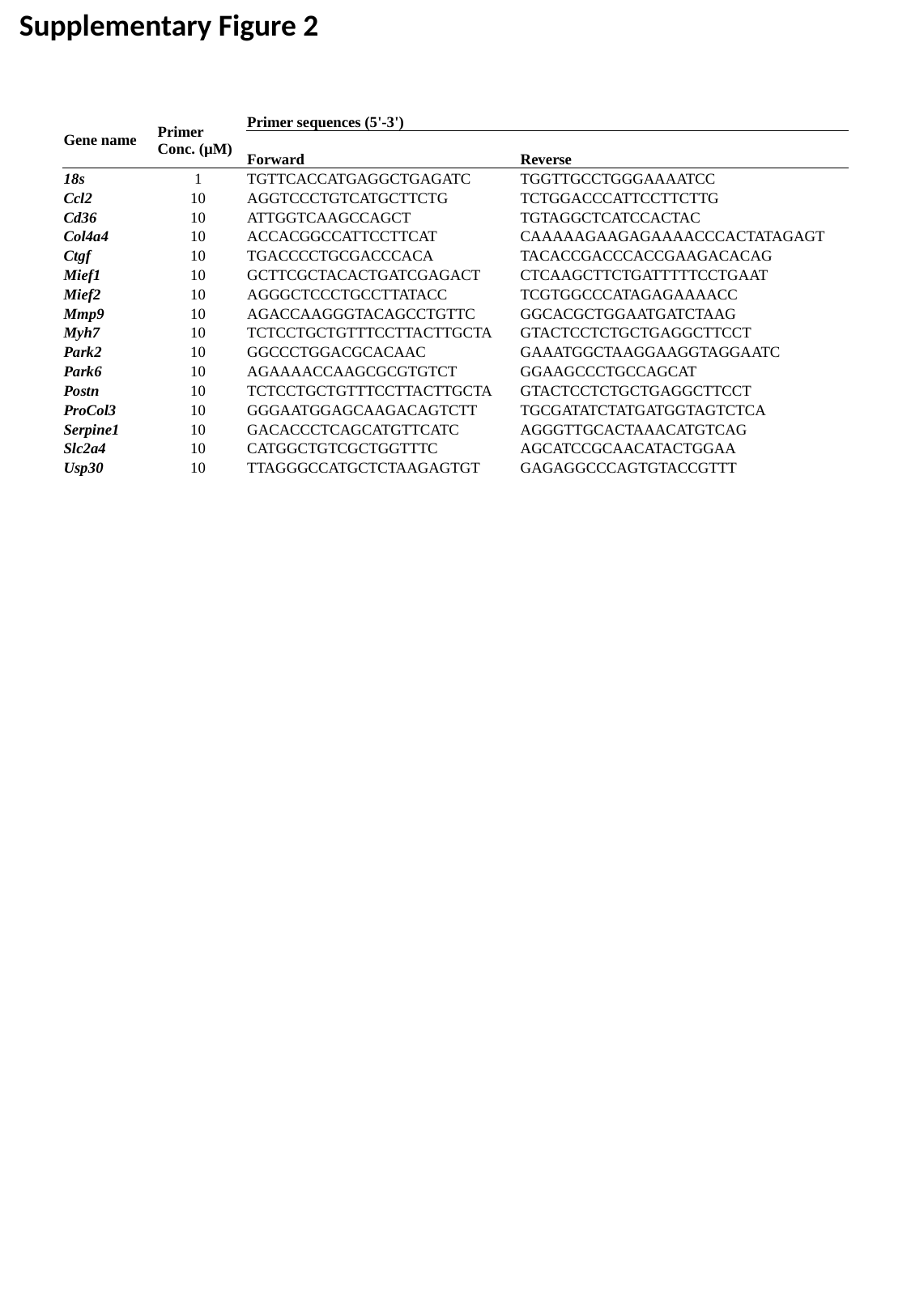

Supplementary Figure 2
| Gene name | Primer Conc. (µM) | | Primer sequences (5'-3') | |
| --- | --- | --- | --- | --- |
| | | | Forward | Reverse |
| 18s | 1 | | TGTTCACCATGAGGCTGAGATC | TGGTTGCCTGGGAAAATCC |
| Ccl2 | 10 | | AGGTCCCTGTCATGCTTCTG | TCTGGACCCATTCCTTCTTG |
| Cd36 | 10 | | ATTGGTCAAGCCAGCT | TGTAGGCTCATCCACTAC |
| Col4a4 | 10 | | ACCACGGCCATTCCTTCAT | CAAAAAGAAGAGAAAACCCACTATAGAGT |
| Ctgf | 10 | | TGACCCCTGCGACCCACA | TACACCGACCCACCGAAGACACAG |
| Mief1 | 10 | | GCTTCGCTACACTGATCGAGACT | CTCAAGCTTCTGATTTTTCCTGAAT |
| Mief2 | 10 | | AGGGCTCCCTGCCTTATACC | TCGTGGCCCATAGAGAAAACC |
| Mmp9 | 10 | | AGACCAAGGGTACAGCCTGTTC | GGCACGCTGGAATGATCTAAG |
| Myh7 | 10 | | TCTCCTGCTGTTTCCTTACTTGCTA | GTACTCCTCTGCTGAGGCTTCCT |
| Park2 | 10 | | GGCCCTGGACGCACAAC | GAAATGGCTAAGGAAGGTAGGAATC |
| Park6 | 10 | | AGAAAACCAAGCGCGTGTCT | GGAAGCCCTGCCAGCAT |
| Postn | 10 | | TCTCCTGCTGTTTCCTTACTTGCTA | GTACTCCTCTGCTGAGGCTTCCT |
| ProCol3 | 10 | | GGGAATGGAGCAAGACAGTCTT | TGCGATATCTATGATGGTAGTCTCA |
| Serpine1 | 10 | | GACACCCTCAGCATGTTCATC | AGGGTTGCACTAAACATGTCAG |
| Slc2a4 | 10 | | CATGGCTGTCGCTGGTTTC | AGCATCCGCAACATACTGGAA |
| Usp30 | 10 | | TTAGGGCCATGCTCTAAGAGTGT | GAGAGGCCCAGTGTACCGTTT |
| | | | | |

## Slide 4
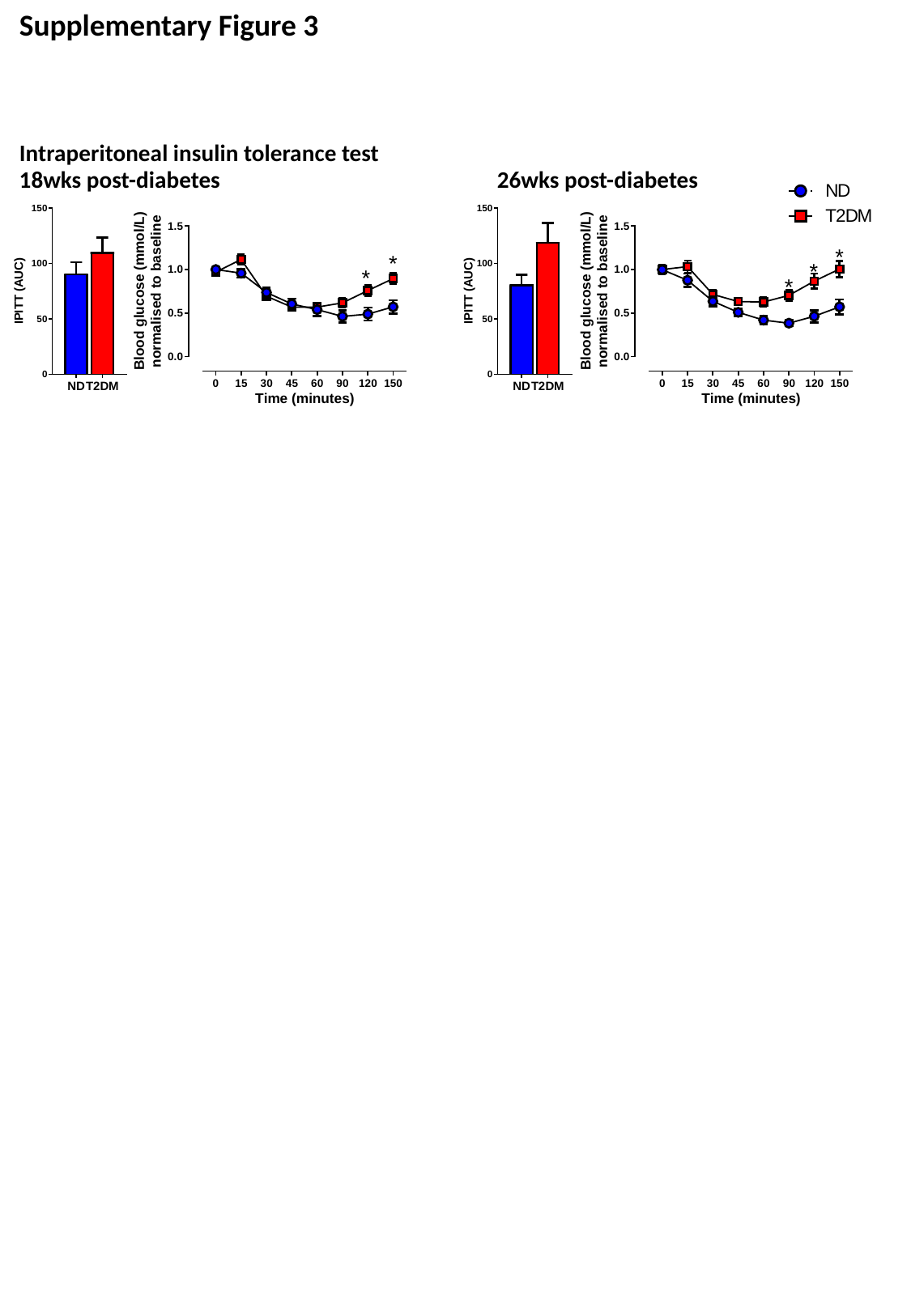

Supplementary Figure 3
Intraperitoneal insulin tolerance test
18wks post-diabetes
26wks post-diabetes

## Slide 5
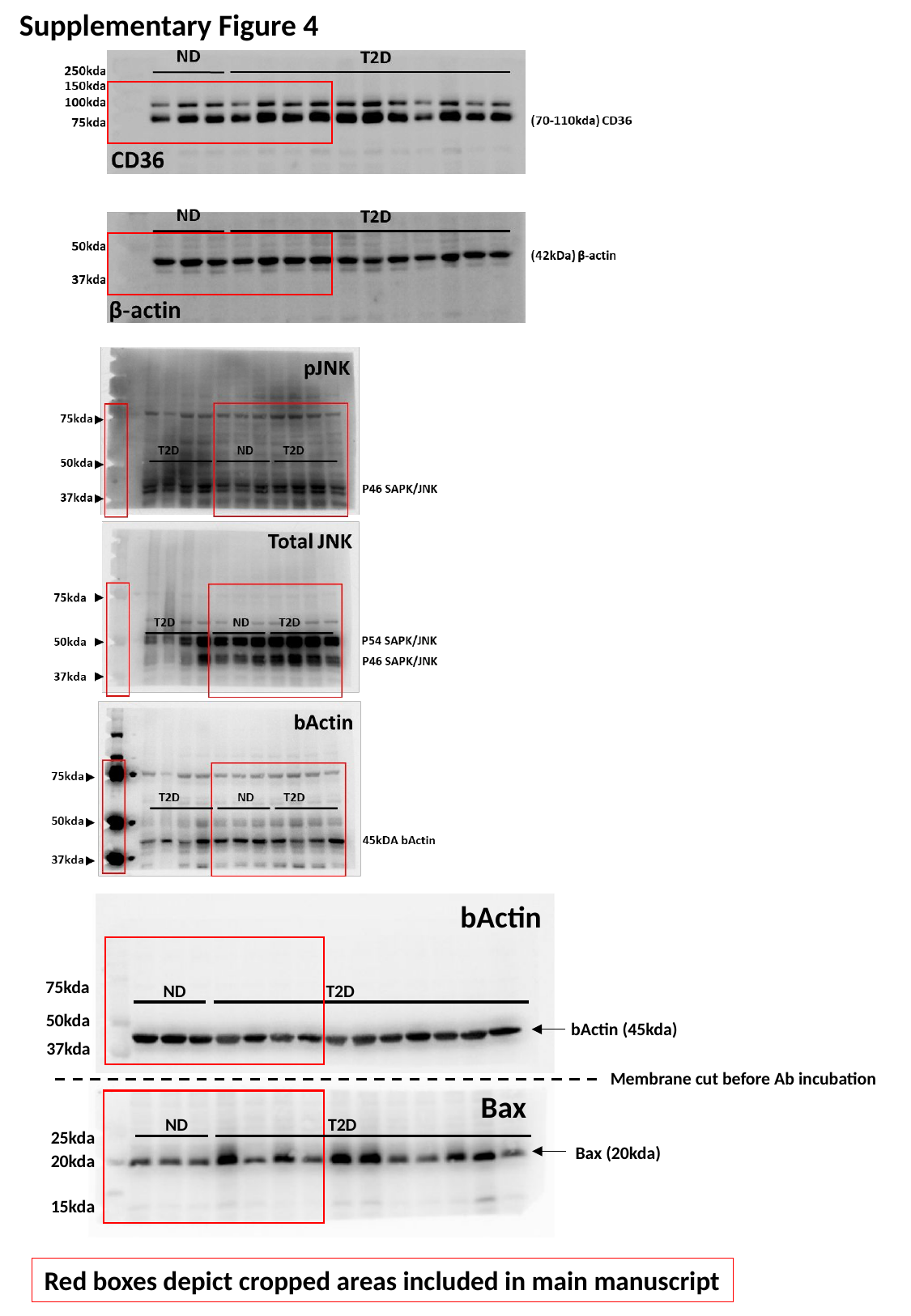

Supplementary Figure 4
bActin
75kda
ND
T2D
50kda
bActin (45kda)
37kda
Membrane cut before Ab incubation
Bax
ND
T2D
25kda
Bax (20kda)
20kda
15kda
Red boxes depict cropped areas included in main manuscript

## Slide 6
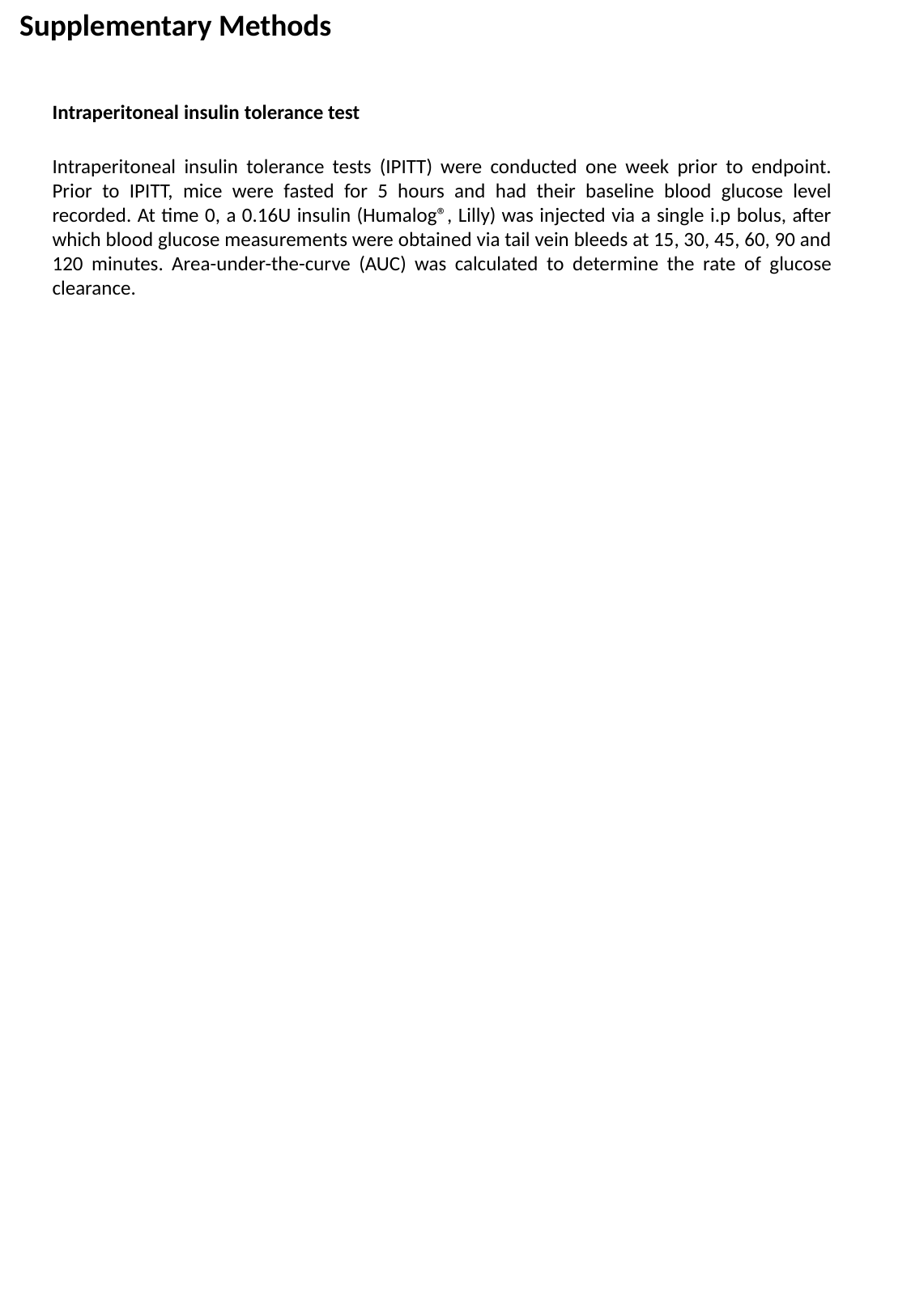

Supplementary Methods
Intraperitoneal insulin tolerance test
Intraperitoneal insulin tolerance tests (IPITT) were conducted one week prior to endpoint. Prior to IPITT, mice were fasted for 5 hours and had their baseline blood glucose level recorded. At time 0, a 0.16U insulin (Humalog®, Lilly) was injected via a single i.p bolus, after which blood glucose measurements were obtained via tail vein bleeds at 15, 30, 45, 60, 90 and 120 minutes. Area-under-the-curve (AUC) was calculated to determine the rate of glucose clearance.
